# Efficacy and Safety of Aprepitant in Allogeneic Hematopoietic Stem Cell Transplantation

**DOI:** 10.1002/phar.1294

**Published:** 2013-05-26

**Authors:** Mayako Uchida, Koji Kato, Hiroaki Ikesue, Kimiko Ichinose, Hiromi Hiraiwa, Asako Sakurai, Tsuyoshi Muta, Katsuto Takenaka, Hiromi Iwasaki, Toshihiro Miyamoto, Takanori Teshima, Motoaki Shiratsuchi, Kimitaka Suetsugu, Kenichiro Nagata, Nobuaki Egashira, Koichi Akashi, Ryozo Oishi

**Affiliations:** 1 Department of Pharmacy, Kyushu University HospitalFukuoka, Japan; 2 Department of Medicine and Biosystemic Science, Kyushu University Graduate School of Medical SciencesFukuoka, Japan; 3 Unit for Cell therapy and Transplantation, Kyushu University HospitalFukuoka, Japan; 4 Center for Cellular and Molecular Medicine, Kyushu University HospitalFukuoka, Japan; 5 Department of Medicine and Bioregulatory Science, Kyushu University Graduate School of Medical SciencesFukuoka, Japan

**Keywords:** aprepitant, vomiting, high-dose chemotherapy, allogeneic stem cell transplantation

## Abstract

**Study Objective:**

To evaluate the efficacy and safety of aprepitant added to standard antiemetic regimens used in high-dose chemotherapy for allogeneic hematopoietic stem cell transplantation (allo-HSCT).

**Design:**

Retrospective medical record review.

**Setting:**

Hematology ward of a university hospital in Japan.

**Patients:**

Of 88 patients treated with high-dose chemotherapy followed by allo-HSCT, 46 received aprepitant and granisetron as antiemetic therapy (between April 1, 2010, and December 31, 2011), and 42 received granisetron alone (between April 1, 2008, and March 31, 2010).

**Interventions:**

Patients in both groups received 3 mg of granisetron intravenously 30 minutes before the administration of anticancer drugs. In the aprepitant group, 125 mg of aprepitant was administered orally 60–90 minutes before the administration of the first moderately to highly emetogenic anticancer drug. On the following days, 80 mg of aprepitant was administered orally every morning. The mean administration duration of aprepitant was 3.3 days (range 3–6 days).

**Measurements and Main Results:**

The primary objective was to evaluate the percentage of patients who achieved complete response (CR; no vomiting and none to mild nausea). The CR rate in the aprepitant group was significantly higher than that in the control group (48% vs 24%, p=0.02). Multivariate analysis showed that nonprophylactic use of aprepitant was associated with failure to achieve CR (odds ratio [OR] 2.92; 95% confidence interval [CI] 1.13–7.99, p=0.03). The frequency of abdominal pain was lower in the aprepitant group (9% vs 25%, p=0.03). Rates of other frequently observed adverse drug events were similar between groups. There was no significant difference in neutrophil engraftment (median 18 vs 17 days), platelet engraftment (median 32 vs 32 days), the incidence of acute graft-versus-host-disease (63% vs 55%, p=0.52), viral infection (74% vs 67%, p=0.49), or 1-year overall survival (63% vs 62%, p=0.90) between the two groups.

**Conclusions:**

The addition of aprepitant to granisetron increases the antiemetic effect without influencing transplantation-related toxicities in allo-HSCT.

Chemotherapy-induced nausea and vomiting (CINV) is one of the most challenging symptoms associated with cancer treatment. To maintain quality of life and enable patients to complete therapy, it is very important to control CINV with the appropriate use of antiemetics.^1^ Dexamethasone in combination with a 5-hydroxytryptamine 3 (5-HT_3_) receptor antagonist represents the standard of care in CINV associated with high-dose conditioning regimens for hematopoietic stem cell transplantation (HSCT).^2^–^4^ Although the combination of dexamethasone plus a 5-HT_3_ receptor antagonist is often used prior to HSCT, vomiting is not controlled in as many as 80–93% of patients.^5^–^13^

Since the neurokinin-1 receptor antagonist aprepitant was approved, the use of triple combination antiemetic therapy (i.e., dexamethasone, 5-HT_3_ receptor antagonist, and aprepitant) has resulted in further improvements in the control of CINV in non-HSCT patients with solid tumors who are treated with moderate to highly emetogenic chemotherapy.^14^–^16^ In addition, aprepitant has been recently reported to prevent CINV associated with high-dose preparative regimens followed by HSCT.^17^–^21^ However, there is still limited information on the safety and efficacy of aprepitant in the setting of allogeneic HSCT (allo-HSCT). Confounding factors that may lead to overlap of acute and delayed CINV in allo-HSCT include the use of calcineurin inhibitors for the control of graft-versus-host disease (GVHD), antibiotics and antifungal agents for infection prophylaxis, total body irradiation (TBI), and high-dose multiday administration of anticancer drugs.^13^, ^22^ Further, many regimens contain drugs such as cyclophosphamide and busulfan that are metabolized by cytochrome (CYP) 3A4. Aprepitant inhibits CYP 3A4,^23^ and concomitant use with CYP3A4 substrates may alter the pharmacokinetic properties of these drugs.^24^–^29^ Thus there is a need to evaluate the antiemetic efficacy and safety of aprepitant in conditioning regimens for allo-HSCT.

In 2010, our institution began using aprepitant as part of the antiemetic prophylaxis strategy for patients undergoing allo-HSCT. The purpose of this study was to evaluate retrospectively the safety and efficacy of aprepitant in allo-HSCT patients.

## Patients and Methods

### Patients

This study was conducted in accordance with the Declaration of Helsinki and its amendments, and the protocol was approved by the Ethics Committee of Kyushu University Graduate School and Faculty of Medicine. Eligible patients were 20 years old and older who underwent myeloablative or nonmyeloablative conditioning regimens (Table 1) followed by allo-HSCT for hematologic malignancies at the Department of Hematology, Kyushu University Hospital. Forty-six consecutive patients received aprepitant and granisetron as antiemetic prophylaxis (the aprepitant group) between April 1, 2010, and December 31, 2011. Forty-two consecutive patients received granisetron alone (the control group) between April 1, 2008, and March 31, 2010, before the introduction of aprepitant. All patients were included in the analysis, and none were excluded because of severe toxicity.

**Table 1:** Schedules of Conditioning Regimens and Antiemetics

Regimens	Dosage and Administration^a^	
TBI/CY		
TBI^a^	2 Gy, twice/day	Days −6, −5, −4
Cyclophosphamide	60 mg/kg, once/day	Days −3, −2
Granisetron	3 mg, twice/day	Days −6 to −2
Aprepitant^b^	Once/day	125 mg on day −3; 80 mg on days −2, −1
BU/CY		
Busulfan	0.8 mg/kg, every 6 hrs	Days −7 to −4
Cyclophosphamide	60 mg/kg, once/day	Days −3, −2
Granisetron	3 mg, twice/day	Days −7 to −2
Aprepitant^b^	Once/day	125 mg on day −7; 80 mg on days −6 to −2
Flu/BU4		
Fludarabine	30 mg/m^2^, once/day	Days −8 to −3
Busulfan	0.8 mg/kg, every 6 hrs	Days −6 to −3
TBI (for UR donor)	2 Gy, once/day	Day −1
Granisetron	3 mg, twice/day	Days −8 to −3, −1
Aprepitant^b^	Once/day	125 mg on day −6; 80 mg on days −5 to −3
Flu/BU2		
Fludarabine	30 mg/m^2^, once/day	Days −8 to −3
Busulfan	0.8 mg/kg, every 6 hrs	Days −6, −5
TBI (for UR donor)	2 Gy, once/day	Day −1
Granisetron	3 mg, twice/day	Days −8 to −3, −1
Aprepitant^b^	Once/day	125 mg on day −6; 80 mg on days −5, −4
Flu/CY		
Fludarabine	25 mg/m^2^, once/day	Days −5 to −1
Cyclophosphamide	30–60 mg/kg, once/day	Days −7, −6
Granisetron	3 mg, twice/day	Days −7 to −1
Aprepitant^b^	Once/day	125 mg on day −7; 80 mg on days −6, −5
Flu/MEL/TBI		
Fludarabine	25 mg/m^2^, once/day	Days −8 to −4
Melphalan	40 mg/m^2^, once/day	Days −3, −2
TBI	2 Gy, once or twice/day	Day −1
Granisetron	3 mg, twice/day	Days −8 to −1
Aprepitant^b^	Once/day	125 mg on day −3; 80 mg on days −2, −1

TBI = total body irradiation; CY = cyclophosphamide; BU = busulfan; UR = unrelated donor; Flu = fludarabine; MEL = melphalan.

a Hematopoietic stem cell transplantation (HSCT) was performed on day 0.

b Patients in the control group did not receive aprepitant.

### Treatments

Intravenous granisetron (3 mg) was started 30 minutes before the administration of anticancer drugs on the first day of preparative regimens prior to allo-HSCT in both groups. Intravenous granisetron was administered every 12 hours while patients received chemotherapy and/or TBI. The aprepitant group received an oral dose of aprepitant (125 mg) 60–90 minutes before the administration of the first moderate to highly emetogenic anticancer regimen. Thereafter, aprepitant (80 mg) was administered in the morning on each chemotherapy day. Aprepitant is approved in Japan for a maximum 5-day course of treatment; therefore, we tailored aprepitant administration schedules to correspond to the severity and duration of CINV associated with each individual conditioning regimen. For example, in the myeloablative busulfan and high-dose cyclophosphamide (BU/CY) regimen, we used aprepitant for 6 days because busulfan (moderate emetic risk) was administered on days −7 to −4, and cyclophosphamide (high emetic risk) was administered on days −3 to −2 (Table 1). Metoclopramide (in most cases) or hydroxyzine was administered for breakthrough nausea or vomiting. Glucocorticoids were not administered for emetic control because most patients were profoundly immunosuppressed and at a greatly increased risk of infection.

For GVHD prophylaxis, either cyclosporine A (1.5 mg/kg IV, every 12 hours) or tacrolimus (0.03 mg/kg/day IV as a continuous infusion) were administered starting on day −1. The target concentrations of cyclosporine A and tacrolimus were 150–200 ng/ml (trough concentration) and 12–15 ng/ml (any timing in a continuous infusion), respectively. The first whole blood concentrations of cyclosporine A and tacrolimus were drawn on day 0. Subsequently, blood concentrations of calcineurin inhibitors were measured almost every day during the early posttransplantation stage. Afterward, dosages of each drug were gradually tapered to achieve lower blood concentrations.

### Data Collection and Assessment

All data were retrospectively collected from the electronic medical record system. The primary end point was overall complete response (CR; no vomiting and none to mild nausea) during the first day of conditioning therapy through 5 days after the end of each conditioning regimen. The average number of days for the conditioning regimens was 6.8 days (range 5–8 days) and 6.6 days (range 5–8 days) in the aprepitant and control groups, respectively.

Secondary end points included the percentage of patients with no vomiting, transplantation-related toxicities, 1-year survival rate, and adverse drug events (ADEs). Nausea and vomiting were graded in four categories: CR, major response (one to two episodes of vomiting and none to moderate nausea or no vomiting and moderate nausea), minor response (three to five episodes of vomiting and any degree of nausea or zero to two episodes of vomiting and severe nausea), and failure (six or more episodes of vomiting with any degree of nausea).^12^, ^20^ Nausea, vomiting, oral mucositis, and ADEs were monitored twice/day (morning and evening) by nurses. The severity of vomiting, oral mucositis, and other ADEs was classified by the Common Terminology Criteria for Adverse Events (CTCAE) v.4.0.

Nurses prepared paper-based ADE monitoring forms excerpted from the CTCAE and used them for the ADE monitoring. Nurses asked patients to self-rate their nausea on a scale from 1 to 4, with 1 indicating no nausea, 2 (mild nausea), 3 (moderate nausea), and 4 (severe nausea). Responses were entered into the electronic medical record system. Engraftment, acute GVHD, and potential infections were evaluated at least once/day by physicians.

Neutrophil and platelet recovery was defined over 3 consecutive days as an absolute neutrophil count of 500 cells/mm^3^ and 50,000 platelets/mm^3^ with no transfusion. Diagnosis and clinical grading of acute GVHD were performed according to established criteria.^30^, ^31^ Overall survival (OS) was defined as time from transplantation to death from any cause. Cyclosporine A and tacrolimus blood concentrations were measured on the day of HSCT and the following 2 days to determine the effect of concomitant aprepitant administration. Calcineurin inhibitor concentrations were expressed as the mean ± SD.

### Statistical Analysis

We used the χ^2^ test or the Fisher exact test to compare categorical data between aprepitant and control groups as appropriate, the Mann-Whitney *U* test to compare age, severity of nausea and vomiting (CR, major response, minor response and failure), and the severity of mucositis, and the *t* test for the concentration of calcineurin inhibitors and time required for engraftment. OS was estimated using the Kaplan-Meier method, and the difference between survival curves was compared by the log-rank test.

To identify the factors associated with non-CR, data on patient characteristics were analyzed by the χ^2^ test or the Fisher exact test. Variables included for univariate analyses were age (50 years or older or 50 years of age or younger), gender, conditioning regimen (myeloablative or nonmyeloablative), and nonprophylactic use of aprepitant. Factors at the p<0.25 level of significance in univariate analyses were evaluated as potential covariates in a stepwise multivariate logistic regression with backward selection. Data were analyzed using JMP v.9.0.0.2 (SAS Institute Inc., Cary NC, USA), and a p value < 0.05 was considered significant.

## Results

### Patient Characteristics

There were no significant differences in gender, median age, or disease between aprepitant and control groups (Table 2). The average number of days of conditioning regimens was 6.8 days (range 5–8 days) and 6.6 days (range 5–8 days) in the aprepitant and control groups, respectively. The average administration duration of aprepitant was 3.3 days (range 3–6 days). The only exception was in the unrelated bone marrow donor source of stem cells that included 50.0% of patients from the aprepitant group versus 23.8% from the control group. The myeloablative-to-nonmyeloablative conditioning regimen ratio was equivalent for both groups. The ratio of recipients treated by the TBI/non-TBI regimen was also equivalent in the aprepitant group (aprepitant vs control: 60.0% vs 59.1% in the myeloablative conditioning regimen with TBI, p=0.952). For GVHD prophylaxis, cyclosporine A with short-term methotrexate (sMTX) was used for related allo-HSCT (aprepitant vs control: 46.7% vs 53.3%, p=0.449) and tacrolimus with sMTX for unrelated allo-HSCT (aprepitant vs control: 55.2% vs 44.8%, p=0.449).

**Table 2:** Patient Characteristics

Characteristics	Control (n=42)^a^	Aprepitant (n=46)^b^	p Value
Gender (%)			
Male	24 (57.1)	28 (60.9)	0.723
Female	18 (42.9)	18 (39.1)	
Age			
Median, yr (range)	47 (22–68)	53 (22–69)	0.224
Diagnosis (%)			
Acute myeloblastic leukemia	17 (40.5)	18 (39.1)	
Acute lymphoblastic leukemia	4 (9.5)	3 (6.5)	
Adult T-cell leukemia/lymphoma	8 (19.0)	6 (13.0)	
Malignant lymphoma	4 (9.5)	11 (23.9)	
Myelodysplastic syndrome	5 (11.9)	3 (6.5)	
Other	4 (9.5)	5 (10.9)	
Conditioning regimen (%)			
Myeloablative regimens	22 (52.4)	20 (43.5)	0.404
TBI/CY	13 (31.0)	12 (26.1)	
BU/CY	4 (9.5)	2 (4.3)	
Flu/BU4	5 (11.9)	6 (13.0)	
Nonmyeloablative regimens	20 (47.6)	26 (56.5)	
Flu/BU2	6 (14.3)	12 (26.1)	
Flu/CY	3 (7.1)	1 (2.2)	
Flu/MEL/TBI	11 (26.2)	13 (28.3)	
Sources of stem cells (%)			
Related donor	16 (38.1)	14 (30.4)	0.504
PB	10 (23.8)	11 (23.9)	
BM	6 (14.3)	3 (6.5)	
Unrelated donor	26 (61.9)	32 (69.6)	
BM	10 (23.8)	23 (50.0)	
CB	16 (38.1)	9 (19.6)	

TBI = total body irradiation; CY = cyclophosphamide, BU = busulfan; Flu = fludarabine; MEL = melphalan; PB = peripheral blood stem cell transplantation; BM = bone marrow transplantation; CB = cord blood stem cell transplantation.

a Patients in the control group received granisetron alone as an antiemetic.

b Patients in the aprepitant group received aprepitant and granisetron as antiemetics.

### Antiemetic Efficacy

**Figure 1:**
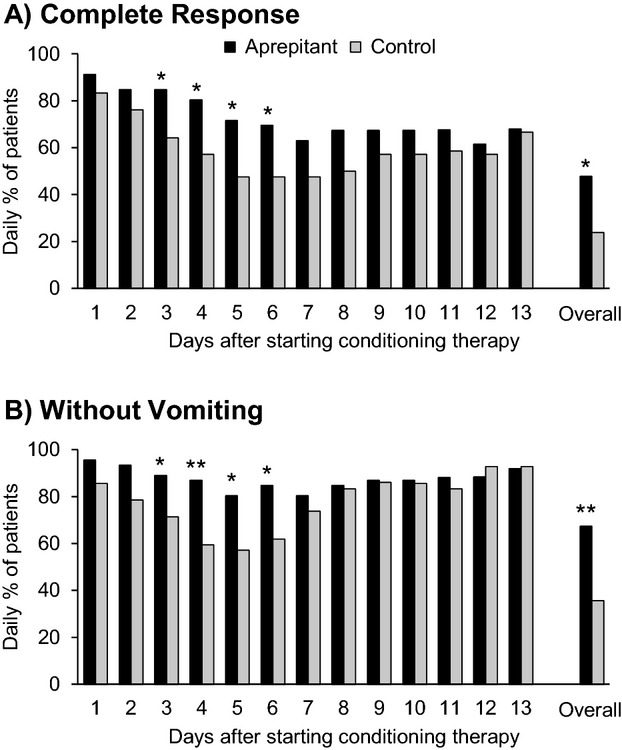
Comparison of the daily and overall percentages of patients with complete response (A) and no vomiting (B) between aprepitant and control groups during the first day of conditioning therapy through 5 days after end of conditioning for each regimen. *p<0.05; **p<0.01.

The overall CR rate in the aprepitant group was significantly greater than in the control group (47.8% vs 23.8%, p=0.019) (Figure 1A). The rates of patients who achieved no vomiting (67.4% vs 35.7%, p=0.003) was also significantly greater in the aprepitant group (Figure 1B). The rates of major response, minor response, and failure in the aprepitant group were 39.1%, 8.7%, and 4.3%, respectively, whereas those in the control group were 23.8%, 33.3%, and 19.0%, respectively (p=0.001). The percentage of patients without nausea was not significantly different than controls (10.9% vs 4.8%, p=0.256). However, the percentage of patients without moderate to severe nausea was significantly lower in the aprepitant group (31.0% vs 52.2%, p=0.044).

The antiemetic effect of aprepitant in patients who received the myeloablative versus the nonmyeloablative conditioning regimen was compared. The percentage of allo-HSCT recipients who achieved CR in the aprepitant group was 30.0% versus 9.1% for the control group (p=0.091), and the rates of CR for the nonmyeloablative conditioning regimen were 61.5% and 40.0%, respectively (p=0.147) (Table 3).

**Table 3:** Complete Responses in Each Subgroup

Conditioning Regimens	Complete response		p Value

	Control (n=42)	Aprepitant (n=46)	
Myeloablative regimens (%)			
TBI/CY	1/13 (7.7)	2/12 (16.7)	
BU/CY	0/4 (0)	1/2 (50.0)	
Flu/BU4	1/5 (20.0)	3/6 (50.0)	
Total	2/22 (9.1)	6/20 (30.0)	0.091
Nonmyeloablative regimens (%)			
Flu/BU2	4/6 (66.7)	6/12 (50.0)	
Flu/CY	0/3 (0)	1/1 (100)	
Flu/MEL/TBI	4/11 (36.4)	9/13 (69.2)	
Total	8/20 (40.0)	16/26 (61.5)	0.147

TBI = total body irradiation; CY = cyclophosphamide, BU = busulfan; Flu = fludarabine; MEL = melphalan.

### Risk Factors for non-CR

In univariate analysis, nonprophylactic use of aprepitant (57.1% vs 31.3%, p=0.027), TBI (39.3% vs 9.4%, p=0.003), and the myeloablative conditioning regimen (60.7% vs 25.0%, p=0.002) were significantly associated with failure to achieve CR. On stepwise logistic regression with backward selection, the predictors significantly associated with failure to achieve CR were nonprophylactic use of aprepitant (odds ratio [OR] 2.92; 95% confidence interval [CI] 1.13–7.99; p=0.031) and myeloablative conditioning regimens (OR 4.62; 95% CI 1.77–13.10, p=0.003).

### Safety

Rates of abdominal pain were lower in the aprepitant group than in the control group (Table 4). The rates for other frequently observed ADEs, such as malaise, diarrhea, headache, skin rash and flushing, and insomnia, were not significantly different between groups. These ADEs were rated as grade 2 or lower and were considered tolerable. Aprepitant was discontinued in one patient each due to ADEs with grade 2 skin rash, grade 1 insomnia, and grade 2 dysuria; however, no patients were excluded from the present study due to severe toxicities.

**Table 4:** Adverse Drug Events

Adverse Drug Events	Control (n=42)^a^	Aprepitant (n=46)^b^	p Value
Malaise (%)	42 (100)	43 (94.1)	0.243
Diarrhea (%)	40 (87.5)	37 (80.0)	0.052
Headache (%)	22 (46.9)	26 (55.9)	0.831
Skin rash and flushing (%)	17 (37.5)	16 (35.3)	0.662
Insomnia (%)	20 (43.8)	14 (29.4)	0.126
Constipation (%)	1 (3.1)	7 (14.7)	0.060
Abdominal pain (%)	12 (25.0)	4 (8.8)	0.025
Hiccups (%)	7 (15.6)	4 (8.8)	0.339
Tremors (%)	3 (6.3)	3 (5.9)	1.000
Hypersensitivity (%)	0 (0)	3 (5.9)	0.243
Dizziness (%)	1 (3.1)	1 (2.9)	1.000
Dysuria (%)	0 (0)	1 (2.9)	1.000
Back pain (%)	4 (9.4)	0 (0)	0.048
Muscle pain (%)	3 (6.3)	0 (0)	0.105

a Patients in the control group received granisetron alone as an antiemetic.

b Patients in the aprepitant group received aprepitant and granisetron as antiemetics.

### Events Associated with HSCT and Clinical Outcomes

**Figure 2:**
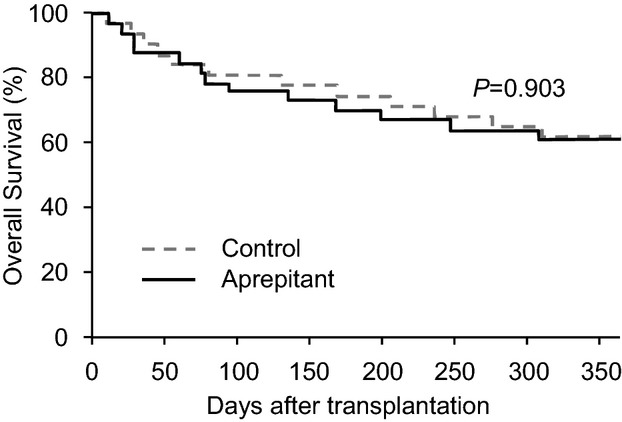
Overall survival in all patients.

The incidence and severity of oral mucositis were not significantly different in the aprepitant and control groups (all grades 15.2% vs 14.3%, p=1.000; grade 2 6.5% vs 4.8%, p=1.000). Neutrophil recovery was obtained promptly in both groups (median 18 days in the aprepitant group and 17 days in the control group, p=0.461). There was no significant difference in the time to achieve platelet engraftment (median 32 vs 32 days, p=0.818). There were no significant differences in the incidence and severity of acute GVHD (all grades 63.0% vs 54.8%, p=0.517; grades 2–3: 34.8% vs 35.7%), bloodstream infection (26.1% vs 23.8%, p=1.000), viral infection (73.9% vs 66.7%, p=0.491), or unexpected fever (43.5% vs 54.8%, p=0.393) between the two groups. In addition, there was no significant difference in OS at 1 year between the two groups (Figure 2).

### Interaction Between Aprepitant and Blood Concentration of Tacrolimus or Cyclosporine A

There were no significant differences in the mean concentration of cyclosporine A in the aprepitant and control groups on day 0 (164.1 ± 79.7 [ng/ml] vs 157.7 ± 57.4 [ng/mL], p=0.789), day 1 (157.1 ± 33.7 vs 170.9 ± 60.7, p=0.432), or day 2 (198.2 ± 45.8 vs 178.4 ± 46.2, p=0.549) (Table 5). Similarly, the mean concentrations of tacrolimus in the aprepitant and control groups were not significantly different on day 0 (13.3 ± 5.6 vs 12.8 ± 5.3, p=0.786), day 1 (13.8 ± 6.4 vs 13.1 ± 3.6, p=0.673), or day 2 (14.3 ± 4.7 vs 14.0 ± 3.4, p=0.799) (Table 5).

**Table 5:** Blood Concentrations of Calcineurin Inhibitors

	Control (n=42)^a^	Aprepitant (n=46)^b^	p Value
Cyclosporine A			
Day 0	157.7 ± 57.4	164.1 ± 79.7	0.789
Day 1	170.9 ± 60.7	157.1 ± 33.7	0.432
Day 2	178.4 ± 46.2	198.2 ± 45.8	0.549
Tacrolimus			
Day 0	12.8 ± 5.3	13.3 ± 5.6	0.786
Day 1	13.1 ± 3.6	13.8 ± 6.4	0.673
Day 2	14.0 ± 3.4	14.3 ± 4.7	0.799

Cyclosporine A and tacrolimus blood concentrations were compared on the day of hematopoietic stem cell transplantation (day 0) and the following 2 days. Calcineurin inhibitor concentrations were expressed as the mean ± SD.

a Patients in the control group received granisetron alone as an antiemetic.

b Patients in the aprepitant group received aprepitant and granisetron as antiemetics.

## Discussion

This study retrospectively evaluated the efficacy and safety of aprepitant in allo-HSCT recipients. No increase in the incidence and severity of ADEs, such as malaise, diarrhea, and headache, occurred in patients who received aprepitant compared with the control group. In addition, there were no significant differences in the incidence of acute GVHD, infectious complications, engraftment, or 1-year OS between the aprepitant and control groups. In contrast, aprepitant dramatically improved the incidence and severity of vomiting and nausea, indicating that aprepitant in addition to standard antiemetic therapies may be a promising antiemetic therapy in the setting of allo-HSCT.

Because aprepitant moderately inhibits CYP 3A4,^23^ drug–drug interactions may occur when aprepitant is administered in combination with anticancer drugs including cyclophosphamide, as well as cyclosporine A or tacrolimus.^24^–^26^ One study^27^ reported that the area under the curve of 4-hydroxy cyclophosphamide decreased by 5% in the presence of aprepitant in patients who underwent cyclophosphamide therapy (1.5 g/m^2^/day × 4 days) for autologous peripheral blood stem cells mobilization, although this was not clinically significant. Another study^28^ reported that aprepitant did not alter the pharmacokinetic profile of melphalan. Recently, we reported that the addition of aprepitant to granisetron improved antiemetic control without influencing ADEs in patients with hematologic malignancies receiving multiday standard-dose chemotherapy. And the regimens in that study were including ESHAP (etoposide + cisplatin + cytarabine + methylprednisolone) therapy for malignant lymphoma,^32^ induction therapy consisted of cytarabine and idarubicin for acute myeloblastic leukemia (AML),^33^ high-dose cytarabine therapy for AML,^34^ induction (cyclophosphamide + daunorubicin + vincristine + prednisolone) therapy for acute lymphocytic leukemia, hyper CVAD (cyclophosphamide + vincristine + doxorubicin + dexamethasone) therapy for malignant lymphoma, or CHOP-V-MMV (cyclophosphamide + vincristine + doxorubicin + etoposide + prednisolone + mitoxantrone + ranimustine + vindesine) therapy for adult T-cell leukemia/lymphoma.^35^ Another study reported that the addition of aprepitant to tacrolimus treatment resulted in modest but clinically insignificant increases in tacrolimus blood concentrations.^36^ Similarly, we found that aprepitant did not affect the blood concentration of cyclosporine A or tacrolimus and did not exacerbate ADEs during the transplantation period. Based on these results, we believe that aprepitant may be safely added to conditioning regimens for allo-HSCT.

The percentage of patients with CR and with no vomiting was significantly higher in the aprepitant group than the control group. Prophylactic use of aprepitant significantly increased the CR rate by 24% (Figure 1). Although methodologic differences preclude direct comparisons between antiemetic regimens with and without aprepitant, similar improvements in the CR rate were reported in a recent clinical trial. A 20prospective, placebo-controlled, randomized phase 3 trial^20^ evaluated the efficacy of aprepitant in combination with ondansetron and dexamethasone in patients treated with ablative conditioning regimens for both auto- and allo-HSCT. The CR rate increased by 16% in the aprepitant group, whereas other outcomes including safety, progression-free survival, and OS were similar in both groups. In that study, approximately half of the patients were auto-HSCT recipients. The levels of tacrolimus in allo-HSCT patients on the day of transplantation were not significantly different. No other subgroup analysis with a focus on allo-HSCT was conducted. Another study^27^ evaluated the pharmacokinetics of aprepitant, concurrent cyclophosphamide, and two major metabolites of cyclophosphamide in patients undergoing HSCT. Although the vast majority of patients underwent allo-HSCT, the effectiveness and safety of the addition of aprepitant to standard antiemetic therapy were not reported in that publication. A randomized double-blind pilot trial of aprepitant added to standard antiemetics during conditioning regimens for HSCT^17^ reported that aprepitant improved emesis prevention without significant changes in cyclophosphamide pharmacokinetics or increased toxicity. Another study administered aprepitant in combination with palonosetron and dexamethasone for patients receiving busulfan and cyclophosphamide therapy prior to allo-HSCT and found that this triple combination was more effective than historical control groups (dexamethasone and either ondansetron or palonosetron) in preventing CINV.^37^ In that study, constipation was similar between groups; no other outcome was evaluated. In the present study, we evaluated the efficacy and safety of aprepitant, as well as the influence of aprepitant on transplantation-related outcomes, in allo-HSCT recipients. Despite the shorter administration duration of aprepitant than in other studies,^20^, ^27^ multivariate analysis clearly revealed that prophylactic use of aprepitant was significantly associated with CR. Therefore, prophylactic use of aprepitant can improve the control of CINV in patients treated with high-dose chemotherapy followed by allo-HSCT.

In a subgroup analysis, the CR rate in the aprepitant group who received the TBI/CY regimen was only 17% (2 of 12) (Table 3). This insufficient control of nausea and vomiting may depend on the duration of aprepitant administration. In fact, 8 of 12 patients (67%) in the aprepitant group who received the TBI/CY regimen already experienced vomiting on day −4 (data not shown). Because aprepitant is not approved for the prevention of radiation-induced nausea and vomiting in Japan,^26^ administration of aprepitant started after TBI (from day −3 to −1) for patients receiving the TBI/CY regimen (Table 1). In animal experiments,^38^ a combination of an NK_1_ receptor antagonist, a 5-HT_3_ receptor antagonist, and dexamethasone completely inhibited kaolin intake, which is an index of nausea and vomiting in mice, induced by 9 Gy of TBI. In a randomized controlled trial,^20^ aprepitant was started on the first day of chemotherapy or TBI and showed benefit in patients receiving high-dose chemotherapy with or without TBI. Taken together, prophylactic use of aprepitant for TBI is likely to improve the control of radiation-induced nausea and vomiting. Moreover, recent American Society of Clinical Oncology guidelines recommend dexamethasone in combination with a 5-HT_3_ receptor antagonist in patients receiving TBI.^2^ Therefore, the addition of dexamethasone is also likely to improve the control of nausea and vomiting in those patients.

Limitations of this study include the retrospective study design, lack of evaluation of specific pharmacokinetic parameters for each chemotherapy agent, and no evaluation of aprepitant for radiation-induced nausea and vomiting with the TBI regimens. Therefore, this study could not confirm that aprepitant affected clinical outcomes of these anticancer drugs and could not confirm the efficacy of aprepitant prior to TBI. Although current guidelines of antiemetic therapy^2^–^4^ recommend dexamethasone in combination with a 5-HT_3_ receptor antagonist for conditioning regimens prior to HSCT, no patient received dexamethasone for CINV prophylaxis in this study. The dose of granisetron was also different in other clinical studies^9^, ^12^ because the approved dose of granisetron is up to 6 mg/day in Japan.^39^, ^40^ Thus our findings cannot be directly applied to other institutions. Palonosetron, a long-acting 5-HT_3_ receptor antagonist, improves the control of CINV in combination with dexamethasone in patients receiving high-dose chemotherapy prior to HSCT.^41^, ^42^ However, because only a single dose of palonosetron is approved in Japan, we used granisetron rather than palonosetron in those patients receiving multiday chemotherapy. Finally, the small sample size in our subgroup analysis precluded definitive conclusions about the antiemetic efficacy of aprepitant.

## Conclusion

The addition of aprepitant to granisetron in conditioning regimens for allo-HSCT increased the antiemetic effect without influencing toxicity and therapeutic outcomes in our patient population. Prospective studies are warranted to further define the role of aprepitant as part of the antiemetic regimen in patients undergoing allo-HSCT.
